# Sarcomeric Gene Variants and Their Role with Left Ventricular Dysfunction in Background of Coronary Artery Disease

**DOI:** 10.3390/biom10030442

**Published:** 2020-03-12

**Authors:** Surendra Kumar, Vijay Kumar, Jong-Joo Kim

**Affiliations:** 1 Department of Anatomy, All India Institute of Medical Sciences, New Delhi 110029, India; surendrakhedarcbt@gmail.com; 2 Department of Biotechnology, Yeungnam University, Gyeongsan, Gyeongbuk 38541, Korea

**Keywords:** sarcomere, dilated cardiomyopathy, left ventricle dysfunction, actin, myosin, troponin, tropomyosin

## Abstract

Cardiovascular diseases are one of the leading causes of death in developing countries, generally originating as coronary artery disease (CAD) or hypertension. In later stages, many CAD patients develop left ventricle dysfunction (LVD). Left ventricular ejection fraction (LVEF) is the most prevalent prognostic factor in CAD patients. LVD is a complex multifactorial condition in which the left ventricle of the heart becomes functionally impaired. Various genetic studies have correlated LVD with dilated cardiomyopathy (DCM). In recent years, enormous progress has been made in identifying the genetic causes of cardiac diseases, which has further led to a greater understanding of molecular mechanisms underlying each disease. This progress has increased the probability of establishing a specific genetic diagnosis, and thus providing new opportunities for practitioners, patients, and families to utilize this genetic information. A large number of mutations in sarcomeric genes have been discovered in cardiomyopathies. In this review, we will explore the role of the sarcomeric genes in LVD in CAD patients, which is a major cause of cardiac failure and results in heart failure.

## 1. Introduction:

Cardiac diseases are one of the main causes of death these days, generally originating as coronary artery disease (CAD) or hypertension. In later stages, many patients may develop left ventricle dysfunction (LVD). Left ventricular ejection fraction (LVEF) is the most determining factor for the prognosis of CAD patients [1,2,3].

LVD is a complex multifactorial condition in which the left ventricle becomes functionally compromised. In the cardiovascular system, the left ventricle plays a central role in the maintenance of circulation because of its role as the major pump in the heart. In the case of LVD, the pumping function of the heart is reduced, leading to symptoms of congestive heart failure (CHF). CAD patients with severe LVD have a higher mortality than those with preserved LV function, and this mortality rate is proportional to the severity of LVD. The rising number of patients with ischemic LVD contributes significantly to the increased morbidity and mortality of cardiac arrests. Impaired pumping by the heart leads to other cardiovascular complications, such as heart failure, myocardial infarction, cardiomyopathies, etc. [3,4,5,6].

LVD includes two distinctive morphologies: hypertrophy and dilation. In LV hypertrophy, ventricular chamber volume remains the same, but the wall of the chamber is thickened. In LV dilation, the chamber volume of the left ventricle gets enlarged when the walls are either normal or thinned. These two conditions are associated with specific hemodynamic variations. In hypertrophic conditions, only diastolic relaxation is impaired, while in the dilated condition, systolic functions are diminished. This results in a change of heart shape from an elliptical to a more spherical form, which causes considerable mechanical inefficiency and deterioration, resulting in CHF [3,6]. Sarcomeric proteins are basic contractile units of the myocyte/myocardium. Several sarcomeric genes have been identified and associated with the pathogenesis of CHF. 

This review will highlight the genetic basis of LVD in the background of CAD. We review the role of common sarcomeric genes (*MYBPC3, TNNT2, TTN, Myospryn*) and their genetic variants with LVD in CAD patients. 

## 2. Common Sarcomeric Protein and Associated Gene Polymorphism

In recent years, remarkable developments have been made in identifying the genetic association of cardiac diseases, resulting in a better understanding of the underlying molecular mechanisms. Several genetic studies have found an association between LVD and dilated cardiomyopathy (DCM). Various sarcomeric protein-encoding genes such as cardiac myosin-binding protein C (MYBPC3), myosin heavy polypeptide, and cardiac troponin I gene mutations, as well as other gene mutations have been identified in DCM [7,8,9,10,11,12,13]. The common sarcomeric gene polymorphisms with their locations and functional roles are given in Table 1.

## 3. Sarcomeric Proteins

**Figure 1:**
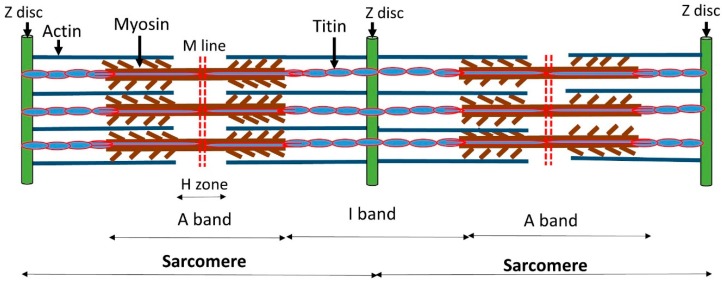
The location and arrangement of the thick and thin myofilament in the sarcomere.

A sarcomere is the functional unit of striated muscle tissue. Skeletal muscles are composed of myocytes formed during myogenesis. Muscle fibers are composed of numerous tubular myofibrils. These myofibrils are a bundle of sarcomeres with repeating units, which appear as alternating dark and light bands under a microscope. Sarcomeric proteins drive muscle contraction and relaxation as these protein filaments slide past each other during these processes. The main components of sarcomere myofilament are actin, myosin, tropomyosin (Tm), and troponin complex (TnT, TnC, and TnI). Myosin protein present in the center of the sarcomere in the form of a thick filament, while actin is thin myofilament and overlaps with myosin. Titin protein‘s C-terminus attaches to M-line, while N-terminus attaches to Z-disc. Titin is anchored to Z-disc by attaching both actin and myosin proteins [24,25,26,27]. During muscle contraction, a conformational rearrangement in the troponin complex is generated by binding calcium ions to TnC, resulting in the movement of Tm, which provides a space for myosin binding on actin, leading to a cross-bridge creation with the help of energy provided by ATP. The contractility of muscles is regulated by calcium ion, which acts on the thin filament’s receptor molecule troponin. Calcium ion is bounded to TnC that successively binds to TnI, releasing it from its inhibitory site on actin [28,29,30,31]. The details are shown in Figure 1.

### 3.1. Myofilament Proteins 

Sarcomeres are in the form of thick and thin myofilament proteins. The thick filament is made up of C protein and myosin, while the thin filament is formed from actin, Tm, and troponin complex. 

#### 3.1.1. Myosin

Myosin is a motor molecule with actin myofilament, and generates force and motion. Myosin consists of two light chains (MLCs) and two heavy chains (MHCs) [32]. Cardiac MHCs have two isoforms in mammals (α and β-isoform) [33]. The α-isoform is related to better actomyosin ATPase activity than β-isoform. Thus, α-isoform has a fast-contractile velocity than β-isoform [34].

MHCs’ isoform expression is sensitive to hormonal changes and cardiovascular stress [35,36]. Isoform shift was observed in human failing myocardium [37,38]. An increase in β-isoform was observed in cardiomyopathy, thyroid depletion, aging, and pressure overload condition [39]. In addition, mammalian myocardium normally primarily expresses α-isoform, but during experimental stimulation of heart failure, it shows upregulation of β-isoform and downregulation of α-isoform [40]. A localized shift of α and β isoforms is noticed in tissues of human ventricles. There is a higher expression of α-isoform in the sub-epicardial than in the sub-endocardial layer [40]. This localized shift of α and β isoforms is consistent with contraction duration and the shorter action potential in the sub-epicardium compared with sub-endocardium [41]. About 80% of atria tissues, present in human ventricular tissues, consist of α-isoform [42]. In atrial fibrillation, the β-isoform expression is approximately doubled [43], and in failing ventricles, α-isoform expression is decreased [42]. In sum, the MHC isoforms’ transition may occur in human atrial and ventricular myocardial disease.

The β-myosin heavy chain (*MYH7*) gene is located on Chr. 14q11.2 and encodes the β isoform in the cardiac myosin heavy chain. Gene mutation in the *MYH7* gene leads to abnormal sarcomeric protein function. The deficiency or altered function of these proteins results in impaired muscle contraction, which results in heart failure [44,45]. 

#### 3.1.2. Actin

Actin is essential for various cell functions. Actin isoforms in mammals are highly conserved. It consists of six isoforms encoded by six different genes. α-skeletal actin, α-cardiac actin, α-smooth actin, and γ-smooth actin are specific in their location and present in skeletal, cardiac, and smooth muscle, respectively. The other two isoforms, β -cyto actin and γ -cyto actin, are universally expressed in tissues [46]. The delicate variations in ratios of actin isoform may lead to alterations in contractility [46]. Additionally, evidence indicates that a reduction in cardiac contractility is associated with decreased expressions of α-cardiac isoform and aberrant expressions of γ-smooth isoform [47]. Humans generally have higher levels of α-skeletal isoform in the heart, compared to rats and mice [48].

#### 3.1.3. Myospryn 

In humans, myospryn protein is a large protein with 4069 amino acid residues (mol. wt. 449 kDa). The C-terminal portion of the protein is made up of approximately 570 amino acids and has tripartite motif (TRIM) proteins-like structure, while the rest of the protein consists of multiple glutamate-rich sequences. Based on this structure, myospryn has also been known as TRIM76 (HGNC Database 2008). Myospryn protein expression is restricted to cardiac and skeletal muscle only [49]. Myospryn protein is localized primarily in a Z-disc of sarcomere below the sarcolemma [50,51].

*Myospryn* gene is situated on Chr 5q14.1 and is related to Z-disc. The *Myospryn* gene is expressed in striated muscle cells, co-localized with the α-actinin sarcomeric protein. In the past, studies have reported an association of *Myospryn* K2906N (rs6859595) polymorphism with left ventricular hypertrophy, cardiac adaptation due to pressure overload [52], and LV diastolic dysfunction in hypertensive patients [23].

### 3.2. Regulatory Proteins

#### 3.2.1. Tropomyosin (Tm)

Tropomyosin is a major regulatory protein, present in a supercoiled form by wrapping around each other—a dimer of two α-helical coil chains. In cells, Tm-isoforms collectively regulate the functional role of actin filaments. Tm-isoforms have two types—(a) muscle Tm-isoforms and (b) non-muscle Tm-isoforms [53]. The interactions between actin and myosin are controlled by these Tm-isoforms and play a pivotal role in controlling Ca^++^sensitive regulation of contraction [54,55,56]. In humans, Tm-isoforms are encoded by four different genes (TPM1, TPM2, TPM3, and TPM4) [57]. α-Tm and κ-Tm from the *TPM1* gene, β-Tm from the *TPM2* gene, and γ-Tm transcribed from the *TPM3* gene are major Tm-isoforms present in human striated muscles. The ratio of β to α-Tm isomer varies from one species to another [58].

The Tm-isoform transition leads to cardiovascular disease. Purcell et al. reported that α-Tm isoform is exclusively expressed by failing heart ventricular muscles [59]. In the heart of chronic DCM patients was found increased expression of κ-Tm-isoform [60,61]. Tm-isoforms regulate cardiac contraction/relaxation, calcium sensitivity, and sarcomeric tension. Tm-isoform shifting potentially affects the overall cardiovascular system.

#### 3.2.2. Troponins

The troponin complex controls the interaction of actin and myosin in striated muscle in response to calcium. This complex contains three regulatory subunits: Troponin-C (TnC; calcium-binding protein), Troponin-I (TnI; inhibitory protein), and Troponin-T (TnT; tropomyosin binding protein).

Troponin-C (TnC): Troponin-C is expressed in cardiac and skeletal muscle and has two isoforms, encoded by genes (*TNNC1* and *TNNC2*). The two isoforms are slow skeleton TnC isoform (ssTnC), encoded by the *TNNC1* gene and expressed in slow muscles and heart muscles; and the fast-skeletal isoform (fsTnC), encoded by the *TNNC2* gene. ssTnC is referred to as cTnC in the heart. The ssTnC/cTnC isoform has been expressed in both developing and adult hearts [55,62]. 

Troponin-I (TnI): Troponin-I has three isoforms: (a) ssTnI (slow skeletal muscle), (b) fsTnI (fast skeletal muscle), and (c) cTnI isoform (cardiac muscle), encoded by *TNNI1, TNNI2,* and *TNNI3* genes, respectively. During development, the ssTnI:cTnI ratio is continuously decreased, and the adult heart has mostly cTnI isoform [63,64]. As the developed heart only expresses cTnI, it does not go through isoform switching under pathological conditions such as DCM, ischemic, and heart failure [65]. In adult transgenic mice, due to increased calcium sensitivity, slow TnI overexpression may impair relaxation and diastolic cardiac function [66]. The cTnI knockout mice show developmental downregulation of ssTnI. TnI depletion changes the mechanical properties of the myocardium. Under relaxed conditions, the myocytes in ventricles show reduced sarcomeres, raised resting tension, and a decreased calcium sensitivity under activating conditions [67]. 

Troponin-T (TnT): The Tn-t protein is present in three isoforms: slow skeletal isoform (ssTnT), fast skeletal isoform (fsTnT), and cardiac isoform (cTnT), encoded by these genes: *TNNT1, TNNT3*, and *TNNT2*, respectively [68,69]. The human heart possesses four common isoforms of cardiac Tn-T (cTnT) i.e., (cTnT1, cTnT2, cTnT3, and cTnT4). A normal adult heart expresses only cTnT3 isoform, while the failing adult heart, as well as the fetal heart, expresses the cTnT4 isoform [70]. Unusual cTnT isoform expressions have been associated with heart ailments. An exon 4 skipped isoform was found highly expressed in failing human hearts [70], and familial HCM human hearts [71]. An over-expression of exon7-excluded cTnT isoform was observed by Craig et al. in a transgenic mouse heart leading to impaired systolic function [72]. The heterogeneous group of TnT isoforms or co-presence of multiple TnT isoforms desynchronize the calcium activation of thin filaments, resulting in cardiac performance reduction [73]. In comparison to wild–type controls, overexpression of one or more functionally different cardiac TnT isoforms in mice resulted in lower left ventricular pressure, declined stroke volume, and slower contractile and relaxation velocities. The author also suggests that co-expression of functionally distinct cTnT isoforms may impair cardiac function in adult ventricular muscle [73].

Troponin T (TNNT2) Gene: TNNT2 gene is located on Chr. 1q32. and encodes a tropomyosin-binding subunit of the troponin complex. This protein is situated on the thin filament of striated muscles and controls muscle contractility in response to Ca^++^ signals. Mutations in the *TNNT2* gene have been positively associated with DCM and familial HCM [20,74,75]. It has been reported that a 5-bp (CTTCT) I/ D polymorphism present in intron 3 of the *TNNT2* gene may impair the skipping of exon 4 and lead to LVD [10,19,20].

### 3.3. Sarcomeric Cytoskeletal Proteins

Cytoskeletal proteins provide mechanical resistance, morphological integrity, and play a major role in maintaining the cell shape of cardiomyocytes. Titin, α-actinin, myomesin, myosin-binding protein C (MyBP-C), and M-protein are the main constituent proteins of this group [76]. 

#### 3.3.1. Titin Protein and Associated Gene Polymorphism

Titin is also known as connectin and encoded by the *TTN* gene, which is located on Chr. 2q31. It is a giant muscle protein of striated muscles that act as a molecular spring and are responsible for passive elasticity. Like a spring, it provides the force to control sarcomere contraction and signaling [22,77,78,79]. Titin is the third most abundant protein in cardiac muscle, after myosin and actin. It spans half of the sarcomere and connects M-line to Z-line. It is the key determinant of myocardial passive tension and plays an important role in the elasticity of cardiac myocytes. These proteins also contribute to the diastolic function of LV filling. Titin consists of two types of protein domains: 1) fibronectin type III domain and 2) immunoglobulin domain. The N-terminal of titin is located in the I-band and connected to Z-disc, and have elastic property. This I-band elastic region has a spring-like PEVK segment, rich in proline, glutamate, valine, and lysine [25,26,27,30,79].

A single gene encodes three major isoforms of titin through alternative splicing [21,79]. Change in ratios of cardiac titin isoforms has been associated with cardiovascular disease. Itoh-Satoh et al. found four possible DCM-associated mutations. The mutated gene expresses a non-functional titin protein that is unable to rotate half sarcomere and decreases binding affinities to Z-line proteins [80]. A familial DCM locus maps to Chr. 2q31 and causes early-onset congestive heart failure (CHF) [81]. RNA binding motif 20 (Rbm20) is a muscle-specific splicing factor, regulating alternative splicing of titin [82]. The Rbm20 knockout rats express a most compliant titin isoform that causes DCM [83,84]. This mutation is associated with the expression of a larger, compliant fetal cardiac titin isoform in severe DCM patients [82].

An 18 bp (TTTTCCTCTTCAGGAGCAA/T) I/D polymorphism falls within the PEVK region that controls the contractile nature of titin. It was previously reported that mutations in the *TTN* gene have been related to different forms of cardiomyopathy, including HCM, DCM, and arrhythmogenic right ventricular cardiomyopathy (ARVC) [9,21,22].

#### 3.3.2. Myosin-Binding Protein C (Mybp-C) and Associated Gene Polymorphism 

MyBP-C is a thick filament-associated striated muscle protein situated in the C zones cross-bridge of A-bands and binds to titin and myosin. MyBP-C and titin collectively form a firm ternary complex, where titin act as a molecular ruler and MyBP-C as a regulatory protein [85,86,87]. In the adult heart, MyBP-C is present in three isoforms in striated muscles, while skeletal muscle expresses only two isoforms. The fsMyBP-C is the fast-skeletal isoform encoded by the gene *MYBPC2* in humans. In humans, MYBPC1 encodes for ssMyBP-C, the slow form of skeletal muscle [88] while *MYBPC3* encodes for human cardiac MyBP-C (cMyBP-C) [89]. In the same sarcomere, fsMyBP-C and ssMyBP-C isoforms can be expressed simultaneously [90] and the diverse arrangements of the specific sarcomere bands are due to the co-existence of fsMyBP-C and ssMyBP-C in variable proportions [91]. The cMyBP-C isoform is found only in cardiac muscle and cannot be trans-complemented by skeletal MyBP-Cs [92]. 

The *MYBPC3* gene located on Chr. 11p11.2 and mutations in this gene were reported in HCM and DCM patients [17,93,94,95]. In 2–6% of Southeast Asian populations, *MYBPC3* 25 bp deletion, located in intron 32 at 3′ region of the gene is noted and associated with a high risk of LVD (left ventricular ejection fraction < 45). This 25-bp intronic deletion results in exon 33 skipping and incorporated missense amino acids at the C-terminal of the protein [14,18]. Incorporation of this mutated protein in myofibrils [14] may cause sarcomere breakdown. Moreover, authors have reported through a protein model that this deletion disrupts the α-helical stretch in the cMyBP-C and an additional α-helix and β-pleated sheets are incorporated in the mutated protein. As the cMyBP-C protein directly binds a subset of Ig domains with titin and myosin through its C8, C9, and C10 domains, any conformational changes in the mutated protein may cause alterations in conformation or direction of the C10 domain. Thus, the inability of myosin binding may have severe effects on sarcomeric organization, suggesting its involvement in the morphological and functional changes of cardiac muscle.

The pathophysiology of cardiac muscles due to truncated and missense MyBP-C has been explained by other mechanisms as well. Due to these mutations, MyBP-C3 mRNA may undergo nonsense-mediated mRNA decay (NMD), which disrupts its proteins through the UPS and may result in cardiac dysfunction [96]. UPS functions also decline with high oxidative stress and the age of an individual. Further, mutated protein may accumulate and disturb cellular homeostasis, and can initiate LVD [97,98]. This deletion polymorphism is also associated with other parameters of LV remodeling, i.e., LV dimensions (LV end-systolic and diastole dimension). This deletion may play an important role in conferring LVD risk in Southeast Asian populations and can be used as an early risk predictor [15]. 

The *MYBPC3* 25-bp deletion polymorphism is quite common with varied frequency in South Asian inhabitants. Studies have suggested that this deletion might have not been present in initial settlers who arrived 50000 to 20000 years ago from Africa, but might have surfaced later in India. The high frequency of this deleterious mutation is somewhat surprising. It has been suggested that with carrier frequency of 2–8% gradation from North to South India, this variation may contribute significantly to the burden of cardiac diseases in the subcontinent [14,99,100].

## 4. Common Sarcomeric Variants Reported with LVD

Studies have reported numerous genetic variants that play a vital role in the pathophysiology of left ventricular dysfunction [15,16,101]. Previously, the *TTN* and *TNNT2* gene variants were studied with various cardiac remodeling phenotypes. Mutation in the *TTN* and *TNNT2* genes were associated with different phenotypes of cardiac remodeling. In intron 3 of the *TNNT2* gene, a 5bp I/D polymorphism is associated with cardiac hypertrophy [20]. The DD genotype is associated with wall thickening of the ventricles and a greater LV mass in the hypertrophy population. Farza et al., however, observed no clinical importance of this variant in cardiac hypertrophy [102]. Rani et al. observed that 5bp deletion in this polymorphism leads to exon 4 skipping at the time of splicing, and is present in a significantly high concentration in HCM patients [19]. Nakagami et al. observed an association between cardiac hypertrophy and myospryn polymorphisms. Authors have reported that in the *Myospryn* gene, AA genotype of K2906N polymorphism plays a risk allele for left ventricular diastolic dysfunction in hypertensive patients [23]. Kumar et al. conducted a case-control study to explore the association of MYBPC3, titin, troponin T2, and myosporin gene deletion polymorphisms. A total of 988 angiographically proved CAD patients and 300 healthy controls were enrolled in this study. Of the 988 CAD patients, 253 were categorized as LVD with reduced left ventricular ejection fraction (LVEF ≤ 45%). The study concluded that there is a significant association of MYBPC3 25-bp deletion polymorphism with elevated risk of LVD (LVEF < 45) (healthy controls v/s LVD: OR = 3.85, *p* value < 0.001; and non-LVD v/s LVD: OR = 1.65, *p* value = 0.035), while the other three studied polymorphisms (Myosporin, TNNT2, TTN) do not seem to play a direct role in LVD as well as CAD risk in north Indians [15,16,103,104]. 

**Figure 2:**
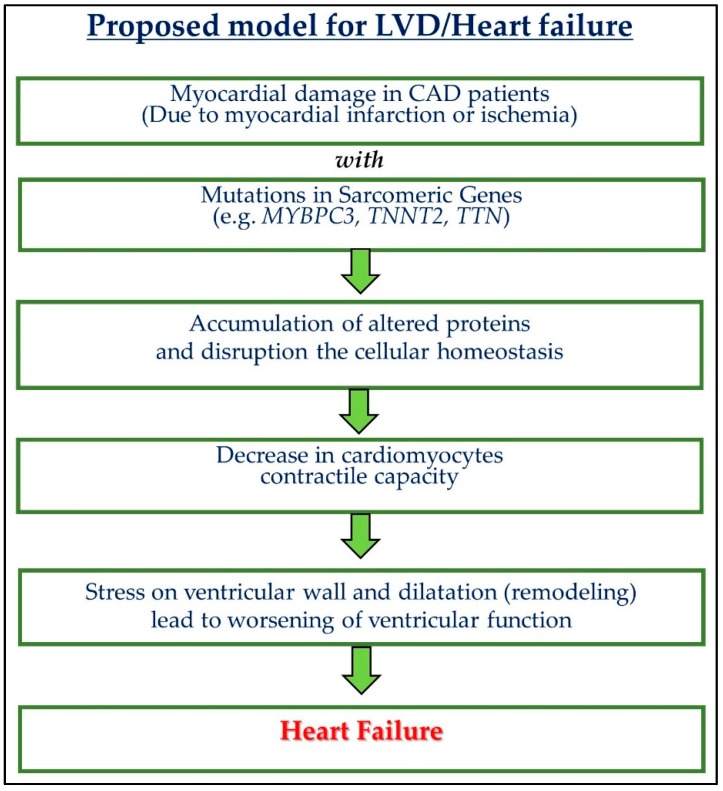
A model for left ventricular dysfunction (LVD)/heart failure. Coronary artery disease (CAD).

Several truncated and missense mutations were reported in MyBP-C, which generates poison peptides and haplo-insufficiency. Due to these mutations, MyBP-C3 mRNA may undergo nonsense-mediated mRNA decay (NMD), which disrupts its proteins through the UPS and may result in cardiac dysfunction [96]. UPS function also declines with high oxidative stress and age. Further, the mutated protein may accumulate and disturb cellular homeostasis and initiate LVD [97,98]. During adrenergic stimulation, cardiac contractility is regulated by MyBP-C. With the help of cyclic AMP-dependent protein kinase and calcium/calmodulin-dependent protein kinase II, MyBP-C goes through reversible phosphorylation [73,105]. A 25-bp deletion in the MyBP-C3 gene causes severe ischemic damage to the cardiac muscle and can develop severe LVD in CAD patients who carry this deletion [15]. In the South Asian population, this 25 bp deletion is relatively common. Analysis of this deletion in different subgroups based on LV ejection fraction (LVEF) shows a significant association of this polymorphism with severe LVD. Patients with LVEF (30–40%) and below 30% have a higher percentage of this deletion genotype. Additionally, this deletion polymorphism is also associated with echocardiogram results such as LV systolic and end-diastolic dimension. To rule out the possibilities of the development of LVD in CAD patients due to confounding factors such as diabetes, smoking, hypertension, and ST-elevation myocardial infarction, the authors performed a multivariate analysis, which shows that the above-stated association was only due to this 25 bp deletion only. This deletion may be responsible, for the development of LVD in CAD patients [15], alone or in combination with hypertension. Based on the above discussion, we proposed a model for left ventricular dysfunction (LVD)/heart failure (Figure 2). 

## 5. Conclusions

It is well established that left ventricular dysfunction is a complex condition, caused by numerous factors—mechanical, neurohormonal, and genetic. Some potential modifiers are shown in Table 2. Sarcomeric genes [15,16], matrix metalloproteinases (MMPs) [106], renin-angiotensin-aldosterone system (RAAS) [101], and inflammatory pathway genes [107] was previously associated with left ventricular dysfunction. In complex diseases, most genetic variants are known to exert minor, but significant effects on disease phenotype. It may be worthy to perform genome-wide association studies to identify novel loci, which may have a vital impact on the development of LVD. Moreover, it is suggested that large, well-designed association studies with functional studies for validation are conducted to establish the combined roles of SNPs in the predisposition and severity of the disease. Moreover, it is important to identify and validate novel mutations in sarcomeric genes using next-generation sequencing and microarrays methods for a complete analysis of genes involved in LVD.

## Figures and Tables

**Table 1:** Common sarcomeric gene polymorphism.

Genes	Location	Type of Polymorphism	Functional Role	Ref.
*MYBPC3*	11p11.2	25 bp Ins/del	*MYBPC3* gene mutation is associated with inherited cardiomyopathies and an increased heart failure risk	[14,15,16,17,18]
*TNNT2*	1q32	5 bp Ins/del	The 5 bp (CTTCT) deletion in intron 3 of the *TNNT2* gene at the polypyrimidine tract was found to affect the gene splicing and branch site selection	[10,19,20]
*TTN*	2q31	18 bp Ins/del	This deletion is present within the PEVK region of *titin* gene that regulates the extensibility of the protein	[21,22]
*Myospryn*	5q14.1	K2906N	This polymorphism is associated with cardiac adaptation in response to pressure overload, left ventricular hypertrophy, and left ventricular diastolic dysfunction in hypertensive patients	[23]

**Table 2:** Potential modifier of Left Ventricular Dysfunction (LVD).

Common Factors	Effect	Ref.
**Environmental Risk Factors**		
Age	Higher in older patients	[108]
Gender	More in men	[109]
Ethnicity	High in African Athletes	[110]
Smoking status	Higher in smoker patients	[111]
Obesity	Higher in obese patients	[112]
Hypertension	Higher in hypertensive patients	[112]
Coronary artery disease	Higher in CAD patients	[112]
Renal disease	Higher in CKD patients	[112]
**Genetic Risk Factors**		
Sarcomeric gene mutations–*MYBPC3, TNNT2, TTN, MYH7, Myospryn*, etc.	↑ ventricular remodeling and LVD	[15,23]
Renin–Angiotensin–Aldosterone System (RAAS) pathway–*ACE* and *AT1* Gene	↑ ventricular remodeling and LVD	[101,113]
Matrix Metalloproteinase (MMPs)–*MMP2, MMP7* and *MMP9*	↑ LVD	[104,106]
Adrenergic pathway–*ADRB1, ADRA2A, ADRB3*	↑ ventricular remodeling and LVD	[103]
Inflammatory pathway–*NFKB1, IL6*, and *TNF-α*	↑ ventricular remodeling and LVD	[104,107]

Coronary artery disease (CAD); Chronic kidney disease (CKD); Left ventricle dysfunction (LVD).
